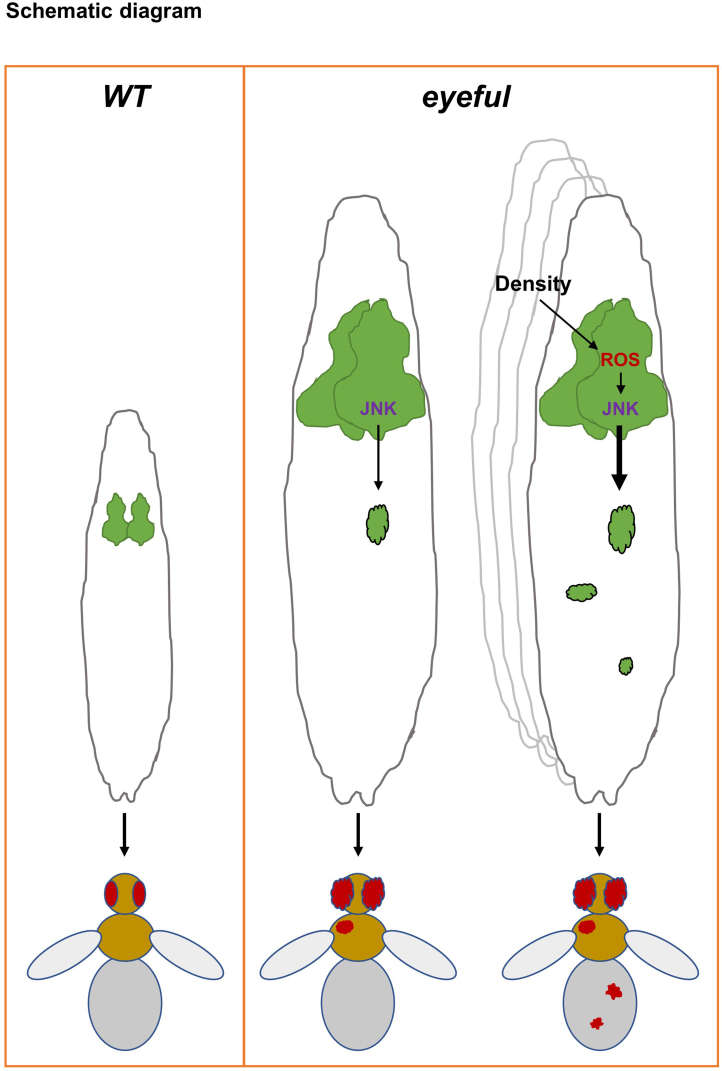# Crowdedness imposes stress on tumor metastasis in *Drosophila melanogaster*

**DOI:** 10.1016/j.gendis.2025.101574

**Published:** 2025-02-27

**Authors:** Wenzhe Li, Zhiyuan Zhang, Lealia Li Xiong, Huiyi Yu, Jiuhong Huang, Ruixiu Cao, Lei Xue

**Affiliations:** a Department of Nuclear Medicine, Shanghai 10th People's Hospital, School of Life Sciences and Technology, Tongji University, Shanghai 200072, China; b College of Pharmacy, International Academy of Targeted Therapeutics and Innovation, Chongqing University of Arts and Sciences, Chongqing 402160, China

Tumor metastasis is the primary cause of mortality in cancer patients, yet its mechanism remains poorly understood. Among the known cancer-related factors, lifestyle and environmental influences such as tobacco use, diet, and viral infections have been considered “stressors”. Prolonged exposure to these stresses significantly increases the risk of tumor formation. Yet, the impact of these environmental factors on tumor metastasis remains an intriguing and open question.

Population density varies greatly between metropolitan and rural areas worldwide. Research has shown that population density significantly affects rodent behavior. In conditions of limited space and frequent unwanted social interactions, rodents exhibit increased stress and aggression, resulting in delayed reproduction in females and intensified aggression in males.^1^^,^^2^ Uncontrolled exponential growth in population density could lead to the extinction of mice.^3^ However, studies on crowding in humans have produced mixed results, as the experience of crowding is influenced not only by physical factors but also by social and psychological factors.

The *Drosophila eyeful* model has been developed to study metastasis *in vivo*. In this system, activation of the Notch pathway in the eyes causes eye enlargement. Further overexpression of Psq and Lola, both Polycomb group epigenetic silencers, leads to the formation of metastatic tumors.^4^ To investigate the relationship between environmental stress and metastasis, we selected crowdedness as an environmental stress and explored its effect on tumor metastasis using the *eyeful* model. Compared with *ey*-GAL4 controls (Fig. 1A–C), *eyeful* flies developed exceptionally large tumorous eyes (Fig. 1B). In some cases, tumor cells migrated to other body parts, such as the head, thorax (Fig. 1D), or abdomen, suggesting that eye tumor cells have migrated to these tissues through metastasis.^5^ Given the pivotal role of the c-Jun N-terminal kinase (JNK) pathway in cancer development, we investigated the activation of JNK signaling in *eyeful* flies. We quantified *puc* transcription in adult flies, and examined *puc*-LacZ expression and phosphorylated JNK (P-JNK) staining in 3rd instar larval eye discs. Compared with the controls, *puc* mRNA level was significantly increased in *eyeful* flies (Fig. 1E). *puc*-LacZ and P-JNK staining (Fig. 1F–J) demonstrated the JNK pathway activation in *eyeful* larval eye discs. These findings indicate that the JNK pathway is activated in *eyeful* tumors.

**Figure 1:**
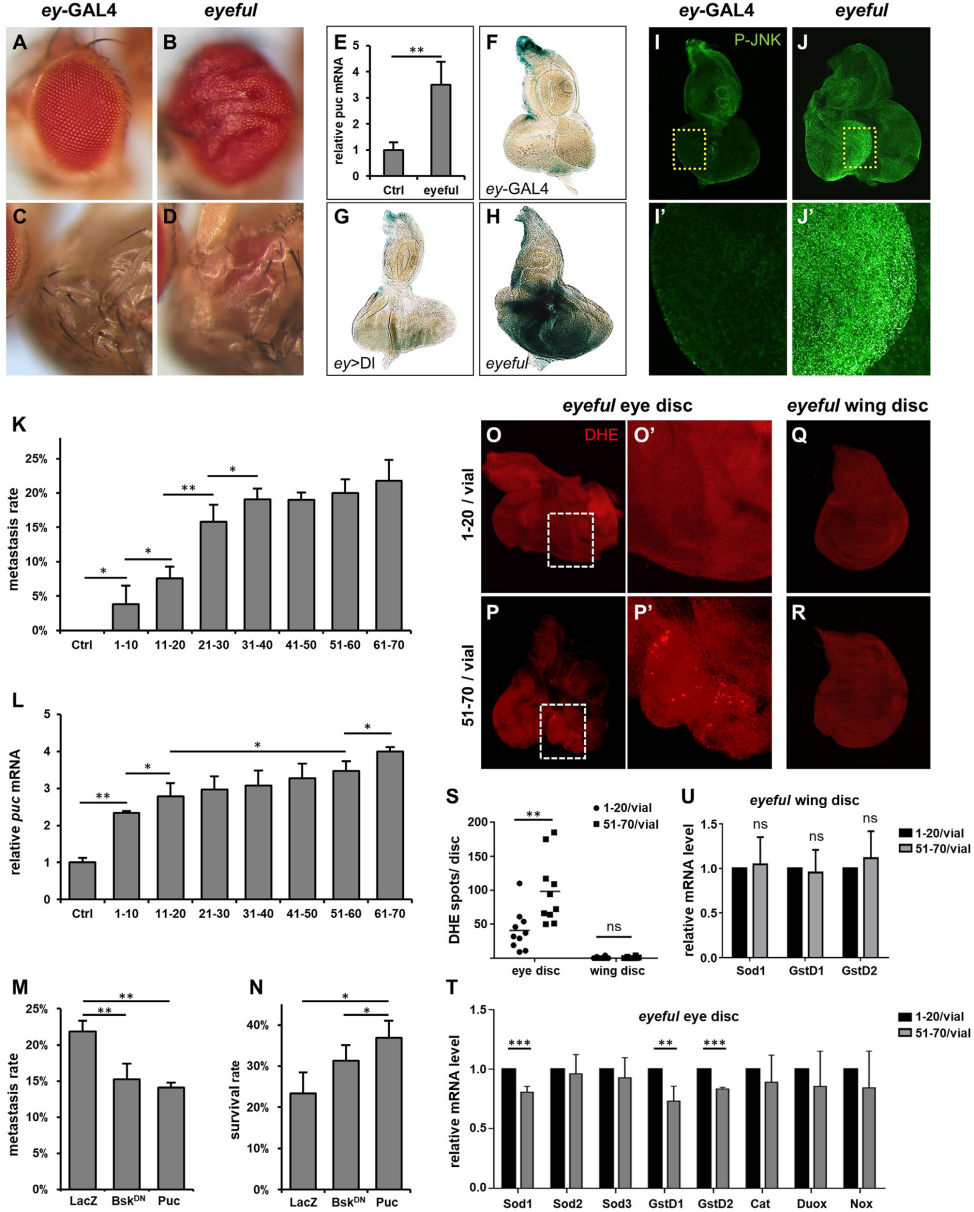
Enhanced ROS-JNK-mediated tumor metastasis in *eyeful* flies under crowded conditions. **(A**–**D)** Compared with the control *ey*-GAL4 flies (A, C), *eyeful* flies exhibited large tumorous eyes (B) with metastatic tumors spreading to other tissues, such as the thorax (D). **(E)** Quantitative reverse transcription PCR analysis revealed a significant increase in *puc* transcription in *eyeful* flies compared with control *ey*-GAL4 flies. **(F–H)** X-gal staining demonstrated enhanced *puc*-LacZ expression in *eyeful* eye discs (H) relative to *ey*-GAL4 (F) and *ey* > Delta (G). **(I, J)** P-JNK staining was notably stronger in the enlarged, folded region of *eyeful* eye discs (J and J′) than in *ey*-GAL4 (I and I′). I′ and J′ are magnified images of the boxed areas in I and J, respectively. **(K, L)** Both tumor metastasis rate (K) and JNK activity (L) in *eyeful* flies increased with rising population density. The *X*-axis in (K) and (L) represents the population density per vial. **(M, N)** Inhibition of JNK signaling by the expression of Bsk^DN^ or Puc reduced metastasis rates (M) and improved survival rates (N) in *eyeful* flies compared with LacZ-expressing flies reared at a density of 61–70 larvae per vial. **(O–R)** Dihydroethidium (DHE) staining of eye discs (O, P) and wing discs (Q, R) from *eyeful* larvae reared in vials containing 1–20 larvae (O, Q) or in vials containing 51–70 larvae (P, R). *eyeful* eye discs (O, P) exhibited higher ROS levels than non-tumorous wing discs (Q, R), particularly under more crowded conditions (P). O′ and P′ are magnified images of the boxed areas in O and P, respectively. **(S)** The statistical data of DHE-positive dot counts from O to R, with *n* = 10. **(T)** The transcription levels of ROS-scavenging genes in *eyeful* eye discs under low- and high-density conditions. **(U)** The transcription levels of *Sod1*, *GstD1*, and *GstD2* in the *eyeful* wing discs cultured under low- and high-density conditions. *p*-values were calculated using a two-tailed *t*-test, and error bars represent ±standard deviation. ∗*P* < 0.05, ∗∗*P* < 0.01, ∗∗∗*P* < 0.001.

To explore the relationship between population density and tumor metastasis, we cultivated *eyeful* larvae at various population densities within standardized vials (each measuring 2.2 cm in diameter and 9.2 cm in height), maintaining a constant volume of 15 mL medium/vial. We found that the metastasis rate increased with population density but reached a plateau at higher densities (Fig. 1K). To assess the impact of developmental factors on the metastasis of *eyeful* tumor, we collected eggs at various densities and quantified the effects of these densities on the larva hatching rate (Fig. S1A), the time to eclosion (Fig. S1B), adult body weight (Fig. S1C), and larval food consumption (Fig. S1D). Our findings revealed no substantial differences in these metrics across the various density groups, suggesting that the increased metastasis rates observed at high population densities are not due to prolonged tumor development or nutritional insufficiencies. These data imply that higher population densities are associated with an elevated risk of metastasis and that crowded environments may elicit stress responses that facilitate the likelihood of metastasis.

Given that JNK signaling is activated by certain stressors and plays a crucial role in tumor metastasis, we postulated that the stress induced by crowding might elevate JNK signaling, thereby contributing to increased metastasis. Consistently, we observed that, compared with controls, *puc* transcription was up-regulated by approximately 2.3-folds in the group with 1–10 larvae/vial, and continued to rise with increasing population density, reaching its maximum in the group with 61–70 larvae/vial (Fig. 1L). *puc* mRNA levels were also increased in the *eyeful* larval eye discs with enhanced density (Fig. S1E). Conversely, *puc* transcription levels in control flies remained unchanged across different population densities (Fig. S1F), suggesting that crowding selectively induces JNK activation in *eyeful* tumors. Since *puc* expression correlates with both population density and metastasis rate, the effect of population density on tumor metastasis is likely mediated through the activation of JNK signaling. To confirm this, we inhibited the JNK signaling in *eyeful* tumors while preserving the same level of crowding stress (61–70 larvae/vial). We found that the expression of a dominant negative form of *Drosophila* JNK (Bsk^DN^) or Puc in *eyeful* tumors markedly reduced the metastasis rate (Fig. 1M), suggesting that metastasis in *eyeful* tumors is reliant on JNK signaling. Moreover, inhibiting the JNK pathway in these tumor-bearing flies substantially improved their survival rates (Fig. 1N).

Persistent JNK signaling is reliant on the production of reactive oxygen species (ROS), which in turn forms a positive feedback loop in cancer cells.^5^ Crowded conditions, acting as a stressor, may activate JNK signaling through the production of ROS. To investigate whether JNK activation induced by crowding in *eyeful* tumors was ROS-dependent, we monitored ROS levels in the eye and wing discs of *eyeful* larvae reared under low (1–20 larvae per vial) versus high (51–70 larvae per vial) density condition. The tumorous eye disc was compared with the tumor-free wing disc, which served as a negative control. The dihydroethidium assay revealed elevated ROS levels specifically in the eye discs, but not in the wing discs, under crowded conditions (Fig. 1O–S), suggesting a positive association between crowding, ROS production, and tumorigenesis. Furthermore, we quantified the transcription of some ROS-related genes in the eye and wing discs of *eyeful* larvae using quantitative reverse transcription PCR. We observed a decrease in the transcription of *Sod1*, *GstD1*, and *GstD2*, while the transcription of *Catalase*, *Sod2*, *Sod3*, *Duox*, and *Nox* remained unchanged, in the eye discs of *eyefu*l larvae exposed to increased crowding (Fig. 1T). Sod1, GstD1, and GstD2 primarily function in the cytoplasm, whereas Catalase, Sod2, Sod3, Duox, and Nox are active in the mitochondria, peroxisome, or extracellular space. This suggests that crowdedness may exert stress by inhibiting the cytoplasmic ROS scavenging system, thereby increasing ROS levels in tumor cells. Further examination of *Sod1*, *GstD1*, and *GstD2* transcription levels in tumor-free wing discs revealed no differences between low and high population densities (Fig. 1U). Based on these observations, we conclude that crowded condition exerts stress to activate ROS in the tumor cells and promotes JNK-mediated tumor metastasis.

In summary, our study reveals that crowdedness, acting as an environmental stressor, exerts selective pressure that accelerates the metastatic rate of tumors. Mechanistically, this effect is mediated through the activation of the ROS-JNK pathway, which may act as a molecular conduit linking external environmental cues to the internal processes governing tumor progression within an organism. These findings suggest that environmental stress could be a pivotal factor shaping the metastatic potential of cancer cells, highlighting the critical need to explore the complex interplay between the environment and the pathogenesis of disease.

## CRediT authorship contribution statement

**Wenzhe Li:** Funding acquisition, Investigation, Methodology, Validation, Writing – original draft, Writing – review & editing. **Zhiyuan Zhang:** Formal analysis, Investigation. **Lealia Li Xiong:** Formal analysis, Investigation. **Huiyi Yu:** Investigation. **Jiuhong Huang:** Investigation. **Ruixiu Cao:** Data curation, Formal analysis, Investigation, Methodology, Validation. **Lei Xue:** Funding acquisition, Supervision, Writing – review & editing.

## Funding

This work is supported by the 10.13039/501100001809National Natural Science Foundation of China (No. 31970536, 32370891) and the Fundamental Research Funds from 10.13039/501100004204Tongji University (No. 2023-3-YB-06).

## Conflict of interests

The authors declared no conflict of interests.

## Figures and Tables

**figs1:**