# Supplementary material for: Crowdedness imposes stress on tumor metastasis in Drosophila melanogaster
Source: Genes Dis. 2025 Feb 27;12(5):101574. doi: 10.1016/j.gendis.2025.101574 (PMC12163375; doi:10.1016/j.gendis.2025.101574)
Supplement: Multimedia component 1 [file mmc1.docx]

**Crowdedness imposes a stress on tumor metastasis in *Drosophila melanoganster***

Wenzhe Li^a,1,^*, Zhiyuan Zhang^a,1^, Lealia Li Xiong^a^, Huiyi Yu^a^, Jiuhong Huang^b^, Ruixiu Cao^a^ and Lei Xue^a,^*

^a^ Department of Nuclear Medicine, Shanghai 10th People's Hospital, School of Life Sciences and Technology, Tongji University, Shanghai, China

^b^ College of Pharmacy, International Academy of Targeted Therapeutics and Innovation, Chongqing University of Arts and Sciences, Chongqing, China

^1^ These authors contributed equally to this work.

*Corresponding Author:

Lei Xue

E-mail: [lei.xue@tongji.edu.cn](mailto:lei.xue@tongji.edu.cn);

Tel: 0086-21-65985407, 0086-13262557721

Wenzhe Li

E-mail: [lwz@tongji.edu.cn](mailto:lwz@tongji.edu.cn);

Tel: 0086-21-65985407, 0086-13661868652

**Supplementary Data**

Supplemental Figure

Materials and methods

Detailed genotypes


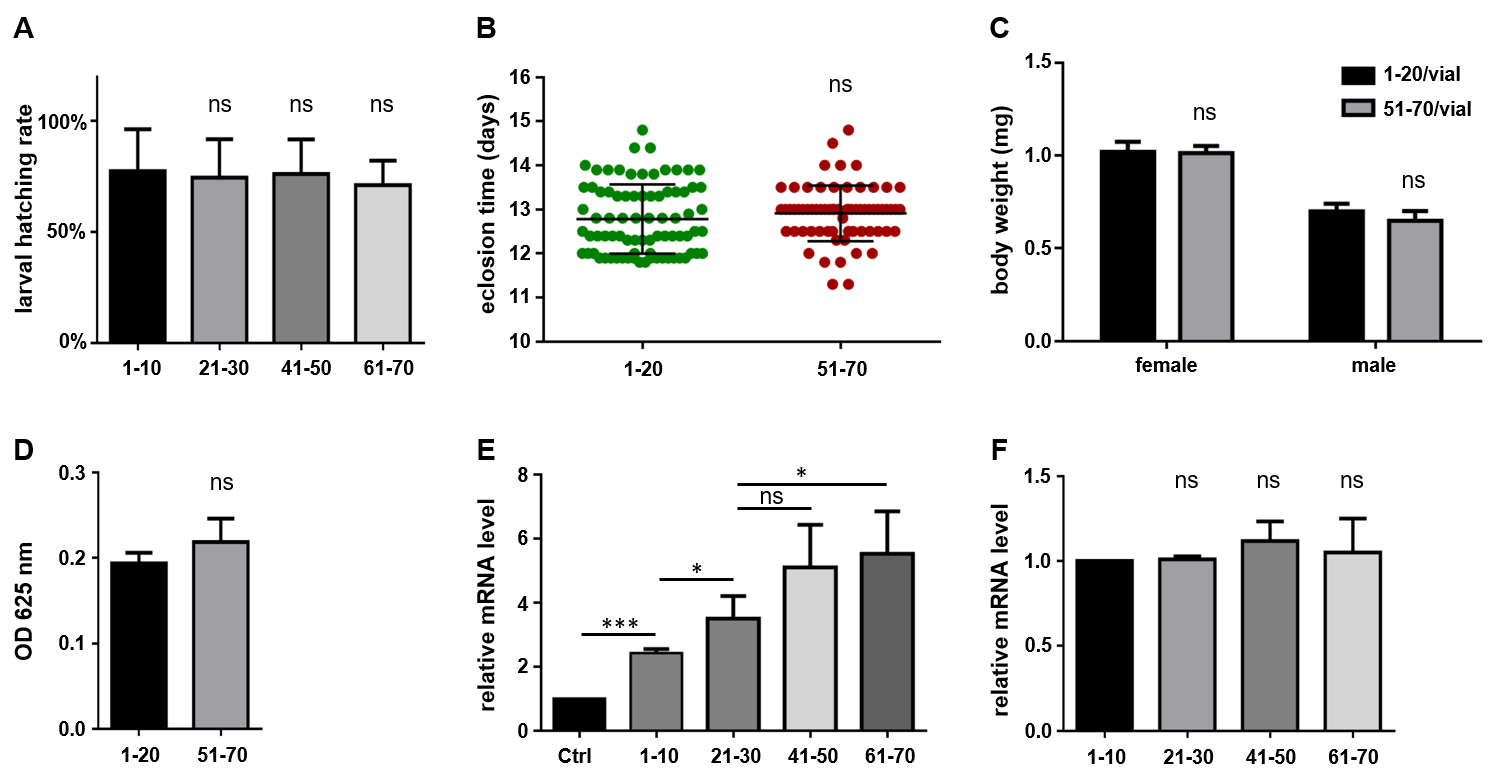
**Supplemental Figure 1**

Figure S1. Development and *puc* transcription of flies under varying culture density.

Illustration of the larval hatching rate (A) and blue dye feeding assay (D), eclosion time (B) and body weight (C) of *eyeful* flies cultured under different density conditions. E shows the *puc* mRNA levels in eye discs dissected from *eyeful* larvae grown under different density conditions. F depicts the *puc* transcription levels of the *ey*-GAL4 control flies reared under various density conditions. Sample sizes for A and B (n≥50), for C and D (n≥30), and for E-F (n≥3). A two-tailed t-test was used to calculate P-values. All error bars represent ± standard deviation (s.d.). * P<0.05; *** P<0.001.

**Materials and methods**

***Drosophila* cultivation and strains**

Flies were reared on a standard culture medium consisting of 85 g corn powder, 65 g brown sugar, 7.5 g agar, 8 g yeast, and 6 mL propanoic acid in 1 L of water. All stocks and crosses were maintained at 25°C. *w^1118^*, *ey*-GAL4, *UAS*-Puc, *UAS*-Bsk^DN^, *puc-LacZ*, *UAS-*LacZ, *UAS-*Dl transgenic strains were obtained from the Bloomington *Drosophila* Stock Center. *eyeful*/*Cyo* were kindly provided by Professor Maria Dominguez.

***Drosophila* crosses**

Males from the *eyeful* stock were employed for all experiments. For the population density study, *eyeful/Cyo* males were crossed with *w^1118^* virgin females. Parental flies were placed in vials containing standard medium at densities of 1, 3, 5, and 10 pairs per vial. Flies were transferred to fresh vials every 12 hours. A minimum of 50 vials were prepared for each density level. The total number of eggs laid per vial was recorded, and after 30 hours of incubation, the number of unhatched eggs and empty eggshells left by hatched larvae were counted to determine larval density. F1 offspring were collected and counted every 12 hours, separated by genotype. *eyeful* flies were further examined for tumor metastasis using microscopy.

**Embryo-to-adult development**

The larval hatching rate was determined by dividing the number of empty eggshells by the total number of eggs laid. The eclosion time was measured from the onset of egg laying to the emergence of adult flies.

**Metastasis Rate Quantification**

Adult flies were anesthetized with carbon dioxide and observed under a microscope for tumor metastasis. The number of metastatic flies in each vial was recorded. At least 50 vials were used for each test. Statistical differences between each pair of groups were determined using the t-test. Error bars represent the standard deviation of the mean.

**X-Gal staining**

Eye discs were dissected from 3^rd^ instar larvae in PBST and stained for β-galactosidase activity.

**Immunostaining**

Immunostaining of imaginal discs was performed using standard protocols. Primary antibody is rabbit anti-phospho-JNK (1:200; Calbiochem #559309), Secondary antibodies is [goat anti-rabbit IgG (H+L), Alexa Fluor 488](https://www.thermofisher.cn/antibody/product/Goat-anti-Rabbit-IgG-H-L-Cross-Adsorbed-Secondary-Antibody-Polyclonal/A-11008) (1:1000; Life technologies A-11008).

**RT-qPCR**

One-day-old adult flies were collected and immediately frozen at -80°C. These flies were then homogenized in 1.5 mL RNase-free microcentrifuge tubes. RNA extraction was carried out using the TRIzol® Plus RNA Purification Kit (Invitrogen), following the standard protocol. For reverse transcription and real-time PCR, Takara Bio Inc.'s PrimeScript™ RT reagent Kit (Perfect Real Time) was utilized, following the SYBR® Green Assay protocol.

To detect the transcriptional levels of *puc* and ROS-related genes, 3rd instar larvae with the genotype of *eyeful*/+ were dissected, and eye and wing discs were collected for RNA extraction and RT-qPCR.

**Body weight**

Adult flies were collected within 8-12 hours after eclosion, separated by sex, and three replicate sets of 10 flies per group were weighed to the nearest 0.01 mg.

**Blue dye feeding assay**

Larvae were provided with food supplemented with 0.5% FCF-blue dye for a period of 90 minutes, washed in ddH_2_O to remove excess dye from their cuticle. Groups of eight larvae were placed into separate 1.5 mL Eppendorf tubes, each containing 500 μL PBS. The samples were homogenized with motorized pestle and mortar, followed by centrifugation at 12,000 RPM for 20 minutes. 100 μL of the supernatant from each sample was transferred to a clear 96-well plate, and the absorbance was measured at 625 nm using a SpectraMax iD3 reader.

**DHE staining**

Eye and wing discs were dissected from 3rd instar larvae in PBS, incubated with 5 μM DHE (N1142; Thermo Fisher Scientific) for 20 minutes in the dark, washed and fixed with 4% PFA/PBS for 30 minutes on ice. After washing with PBS, samples were imaged immediately using an Olympus Fluorescence microscope.

**Detailed genotypes**

**Figure 1 Detailed genotypes**

(A, C) *w; ey*-GAL4*/+*

(B, D) *w; ey-*GAL4 *UAS-*Psq+Lola *UAS-*Dl/+

(E) *w; ey-*GAL4*/+*

*w; ey-*Gal4 *UAS-*Psq+Lola *UAS-*Dl/+

(F) *w; ey*-GAL4*/+; puc-lacZ/+.*

(G) *w; ey*-GAL4/*UAS-*Dl*; puc-lacZ*/+

(H)  *w; ey-*GAL4 *UAS-*Psq+Lola *UAS-*Dl/+*; puc-lacZ*/+

(I, I’) *w; ey*-GAL4*/+*

(J, J’) *w; ey*-Gal4 *UAS-*Psq+Lola *UAS-*Dl*/+*

(K, L) *w; ey*-GAL4/+

*w; ey*-GAL4 *UAS-*Psq+Lola *UAS-*Dl*/+*

(M, N) *w; ey*-Gal4 *UAS-*Psq+Lola *UAS-*Dl/+; *UAS*-LacZ/+

*w; ey*-Gal4 *UAS-*Psq+Lola *UAS-*Dl/+; *UAS*-Bsk^DN^/+

*w; ey*-Gal4 *UAS-*Psq+Lola *UAS-*Dl/+; *UAS*-Puc/+

(O-U) *w; ey-*GAL4 *UAS-*Psq+Lola *UAS-*Dl/+

**Figure S1 Detailed genotypes**

(A) *w; ey*-GAL4/+

*w; ey-*GAL4 *UAS-*Psq+Lola *UAS-*Dl/+

(B-E) *w; ey-*GAL4 *UAS-*Psq+Lola *UAS-*Dl/+

(F) *w; ey*-GAL4/+
